# Identifying patient‐centered outcomes in progressive familial intrahepatic cholestasis: Results from IMPACT

**DOI:** 10.1002/jpn3.70455

**Published:** 2026-05-21

**Authors:** James E. Squires, Gitta H. Lubke, Emily Ventura, Alexandra Perez, Paula M. Hertel, Emily R. Perito, Laura Bull, Melissa Kochanowsky

**Affiliations:** ^1^ Division of Gastroenterology and Hepatology UPMC Children's Hospital of Pittsburgh Pittsburgh Pennsylvania USA; ^2^ PFIC Network Stanton Kentucky USA; ^3^ University of Notre Dame South Bend Indiana USA; ^4^ Division of Pediatric Gastroenterology and Hepatology Texas Children's Hospital Houston Texas USA; ^5^ Division of Gastroenterology and Hepatology UCSF Benioff Children's Hospital San Francisco California USA; ^6^ Department of Medicine, Division of Gastroenterology and Hepatology, UCSF, Liver Center Institute for Human Genetics San Francisco California USA

**Keywords:** comparative effectiveness research, genetic cholestasis, patient reported outcomes, treatment experience app (TEA)

## Abstract

**Objectives:**

In rare diseases like progressive familial intrahepatic cholestasis (PFIC), lack of evidence results in treatment variability and strategies for families to engage care teams to understand how variability affects overall health are unknown. Patient‐centered comparative effectiveness research (CER) has been identified as an approach to address these challenges, but CER‐specific knowledge and infrastructure are lacking. We present the results from IMPACT (Identifying research targets by Merging Patient And Clinician Treatment information), a 2‐year capacity‐building project for patient‐centered CER.

**Methods:**

Six learning modules were adapted from the Patient‐Centered Outcomes Research Institute to teach participants about CER and elicit feedback for patient‐specific CER priorities and processes in PFIC. Participants completed modules and engaged in focus groups (12 virtual and in‐person focus groups). Engagement quality was assessed using a validated patient engagement evaluation tool.

**Results:**

Participation in learning modules varied between 16 and 31 patient/family participants (mean 22), and 5 and 8 clinicians/researchers (mean 5.3). Focus group participation varied between 7 and 35 patient/family participants (mean 13.1) and 3–35 clinicians/researchers (mean 7.3). Collated data enabled develop of (1) the treatment experience app (TEA): a web‐based, community‐built information tool to display relevant, research‐based, lay language therapeutic approaches, augmented by patient experience information and (2) the IMPACT roadmap, a manual for future PFIC patient‐centered research. Engagement assessments demonstrate consistently strong and steadily improving levels of engagement.

**Conclusions:**

IMPACT successfully engaged key stakeholders to build shared understanding of patient‐centered CER, define PFIC‐specific CER targets, and develop strategies for equitable and sustainable patient–researcher partnerships.

## Abstract (highlights)

**What is Known:**

- In progressive familial intrahepatic cholestasis (PFIC), a lack of clinical guidelines results in treatment variability and little evidence of how different approaches affect outcomes that are important to patients.
- Patient‐centered comparative effectiveness research (CER) has been utilized as a strategy to address these shortcomings in other diseases, but CER‐specific knowledge and infrastructure are lacking in PFIC.

**What is New:**

- IMPACT (Identifying research targets by Merging Patient And Clinician Treatment information) engaged the PFIC community to provide a knowledge base for the development of patient‐centered CER tools.
- The treatment experience app and the IMPACT roadmap represent novel, co‐developed patient‐centric resources to perform impactful future patient‐centered CER.
- IMPACT represents adaptable and scalable approaches for other rare disease networks.

## Graphical abstract

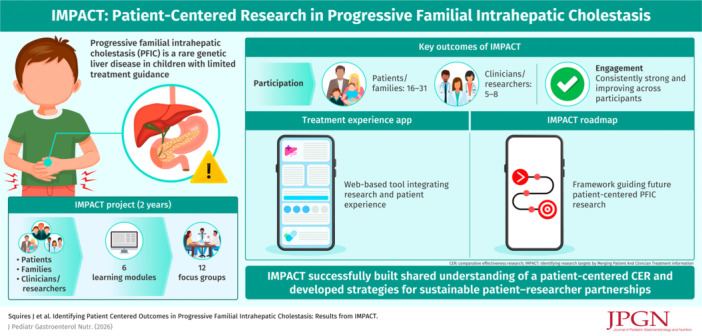

## 1 INTRODUCTION

Progressive familial intrahepatic cholestasis (PFIC) is a group of rare genetic disorders related to defects in bile transport that can lead to liver failure during childhood. PFIC diseases exhibit considerable intra‐ and inter‐subtype variability in both severity and prognosis. Treatment is challenging, and with no definitive medical therapies available efforts often start with symptom management but progress to surgical interventions, including biliary diversion and liver transplant.^1^, ^2^, ^3^, ^4^

As with many rare diseases, there are limited guidelines for the management of PFIC disorders. Existing documents are based mainly on expert opinion, underscoring the lack of robust evidence to navigate the clinical course.^5^ As a result, patients and families are often left fully dependent upon their individual providers to make complex care decisions regarding treatments and interventions. Critically, how patient values, priorities, and goals are incorporated into these decisions is entirely unknown, and efforts to identify patient‐driven objectives within current treatment and research programs have been lacking.

The PFIC Network (PN) is a patient advocacy organization committed to improving the lives of PFIC patients and families while striving for a cure for PFIC and related diseases. Founded in 2018 by caregivers as a novel organization providing resources and support, PN has gained international experience in collaboratively engaging patients and stakeholders to produce disease education materials, create a community for support and advocacy, organize hybrid Family & Scientific Conferences, host the PFIC Network Patient Registry (PNPR, http://www.pfic.org/get-involved/pfic-patient-registry/), and drive research.^6^

In April 2022, PN convened a 2‐day meeting with broad stakeholder representation to identify fundamental themes for PFIC families that could be a focus for PN. During the conference, PFIC caregivers repeatedly expressed that they felt uninformed in their treatment journeys—not knowing what the best “next step” was for their children and their family—and unclear about how to engage care teams to address these anxieties. Caregivers’ sentiments were compounded by limited availability of unbiased information about various treatments in lay language and a dearth of knowledge about how these treatments benefited or impacted their child's clinical courses. One strategy identified to leverage PN's focus on partnership between patients and providers, and to address these challenges was patient‐centered research and specifically patient‐centered comparative effectiveness research (CER). Patient‐centered CER is a specific type of research that has been utilized minimally in rare diseases and never previously in PFIC. It specifically aims to help patients, or their caregivers, communicate and make informed healthcare decisions, amplifying their voices in assessing the value of healthcare options.^7^

Because patient‐centered CER experience was extremely limited among both PN providers and families, we developed and implemented IMPACT (Identifying research targets by Merging Patient And Clinician Treatment information), a 2‐year capacity‐building project for patient‐centered CER. Our aims were to build sustainable expertise and supportive infrastructure for PFIC‐specific patient‐centered CER and to identify key points along the PFIC treatment journey that can be targeted by future patient‐centered CER—both novel efforts in PFIC.

## 2 METHODS

### 2.1 Ethics statement

The data collection strategy, consents/assents, and all individual surveys were submitted and approved by North Star Review Board (https://learningirb.org)—NB300131. North Star Review Board is an independent non‐profit Research Ethics Review Board (RERB).

### 2.2 Learning/Feedback modules

IMPACT (Identifying research targets by Merging Patient And Clinician Treatment information) was developed as a collaboration between PN (https://www.pfic.org), a parent‐led PFIC advocacy group (E.V., M.K., W.P., and G.H.L.), and the Starzl Network for Excellence in Pediatric Transplantation (SNEPT, starzlnetwork.org), a multi‐center learning health system dedicated to pediatric liver transplant (E.R.P. and J.E.S).^8^, ^9^ IMPACT was funded by a Capacity Building Engagement Award (award period July 1, 2023 to June 30, 2025) secured by PN from the Patient Centered Outcomes Research Institute (PCORI, EOSO‐40455); PCORI's intent with these awards is to support engagement of stakeholders who are not trained as researchers in CER efforts and to prepare patients/families and researchers to be effective partners on these teams. With this dual goal in IMPACT, our objective was to build sustainable expertise and supportive infrastructure for CER into PN and the PFIC community.

Learning/feedback modules previously developed by SNEPT^7^ were adapted by project leads to be PFIC focused for IMPACT (M.K., G.H.L., revised by J.E.S., A.P., E.V., E.R.P.). Each module (1) provided participants with background information about IMPACT, (2) taught participants about concepts of patient‐centered CER, using brief videos and text adapted from PCORI's Research Fundamentals course,^10^ and then (3) elicited their ideas for CER priorities and processes in IMPACT (Supporting Information S1: Figure S1). Modules were available in both “patient partner” (i.e., patient/parent/caregiver/PFIC family member) and “provider” (i.e., clinician/researcher/healthcare provider) versions, delivered as self‐guided lessons/surveys (15–20 min per module), and released sequentially over the 2‐year project period. Overall language targeted a 5th–6th grade reading level (Flesh‐Kincaid) with participants reporting minimal demographics, completing modules, and then answering multiple‐choice and open‐ended survey questions. All stakeholders for the PN community were invited by distributing the module invitations through the PN email listserv and by PN physician stakeholders directly to their patient partners and colleagues via email. Additionally, modules were made available via the PN website for access and participation. REDCap was utilized and hosted at PN's account with Amazon Web Services. This data collection tool permits separation of personal identifiers from survey responses. Subsequent survey and focus group interview guides were developed by the researchers (M.L. and G.H.L.) and reviewed and pilot‐tested by patient and clinical partners (J.E.S. and A.P.) prior to broader community participation. Participation in all IMPACT activities was solicited via PFIC Network contact lists and multiple social media campaigns; as a result, exact response rates (i.e., percentage of people who participated out of those who were invited) could not be calculated. Actual participation numbers for the learning/feedback modules and focus groups are presented in the Supporting Information S1.

### 2.3 Evaluation tools

To assess engagement quality and refine implementation over the award period, we used continuous evaluation tools—including the widely used and validated patient engagement evaluation tool (PEET)^11^ and internally developed end‐of‐year 1 and end‐of‐project surveys (Supporting Information S1: Section S1).

### 2.4 Treatment experience app (TEA) development

**Figure 1:**
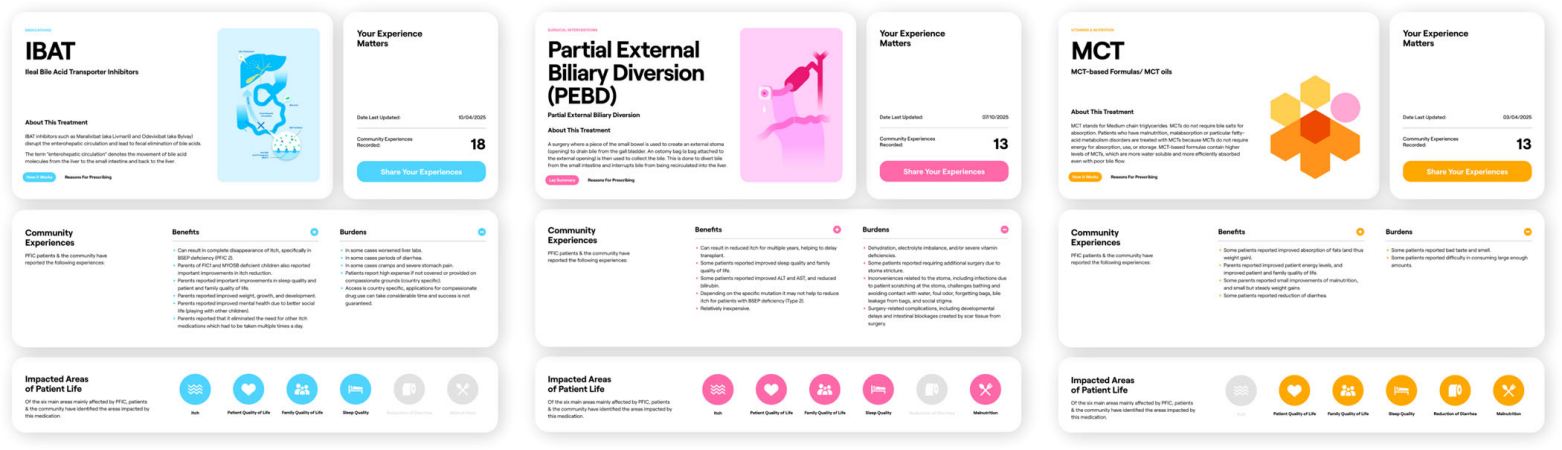
TEA visualizations. Representative sections from each treatment pathway (medications, surgeries, vitamin/nutrition) demonstrating visualizations of the TEA. Overview sections (top left) provide information about therapy and mechanisms of action. “Community experiences” section highlighting both the “benefits” and “burdens” of each therapy as experienced by the PN community (middle section) as well as “impacted areas of life” (bottom section) are shown. The number of entered experiences is shown (top right). PFIC, progressive familial intrahepatic cholestasis; PN, PFIC Network; TEA, treatment experience app.

During the first 12 months of IMPACT, the primary focus was on the development and launch of the TEA. The platform was created through an iterative, user‐centered design process that included data gathered from module surveys, focus group discussions, and feedback sessions (Figure 1). Key features included open text fields that allow participants to describe symptoms, side effects, and treatment outcomes in their own words. Patient‐level experiences can be entered into the TEA via a REDCap secure data repository with information collected about demographics, treatment type, length of treatment, and overall experience (side effects, impact on quality of life, insurance barriers, etc.) (Supporting Information S1: Section S2). Clinical terminology was translated into lay language through a combination of plain‐language guidelines, medical expert review, and readability testing to ensure accessibility across varying literacy levels. Data are stored securely with personally identifying information removed and are organized into a searchable user‐friendly interface to support patients in understanding treatment options and learning from the experiences of others.

### 2.5 IMPACT patient‐centered CER roadmap

Quantitative and qualitative data from all feedback surveys and focus group discussions were analyzed and summarized to create the IMPACT Roadmap, a manual for future patient‐centered CER in PN. Roadmap development resulted from engagement of all stakeholders to define priorities, develop tools, describe methodological considerations, and identify strategies for patient engagement and dissemination.

### 2.6 Statistical analysis

Quantitative data on participant activity and from participant evaluations were analyzed using descriptive statistics. Qualitative data from open‐ended questions on the feedback surveys and transcripts from audio recording of the focus groups were analyzed using a grounded theory approach (M.K. and G.H.L.). Themes were derived from the data guided by researcher notes (M.K., J.E.S., G.H.L. and A.P.) made during all meetings and focus groups; data saturation was discussed for each section by the research team prior to finalizing.

## 3 RESULTS

### 3.1 IMPACT participants

The number of participants in each learning module varied between 16 and 31 patient/family participants, and between 5 and 9 clinician researchers. Focus group participation varied between 16 and 29 patient/family participants and 3–8 clinicians/researchers. The majority of the focus groups were held virtually, with only two focus groups held in‐person (coinciding with PN conferences). The in‐person focus groups at the two conferences had substantially higher attendance, 35 patient/family and 35 clinicians at the first meeting and 16 patient/family participants with 11 clinicians at the second meeting (Supporting Information S1: Section S3). Apart from one patient under the age of 18 who participated in all modules and focus groups, and one adult patient over the age of 18, the patient experience was represented by parents or caregivers.

### 3.2 TEA

**Figure 2:**
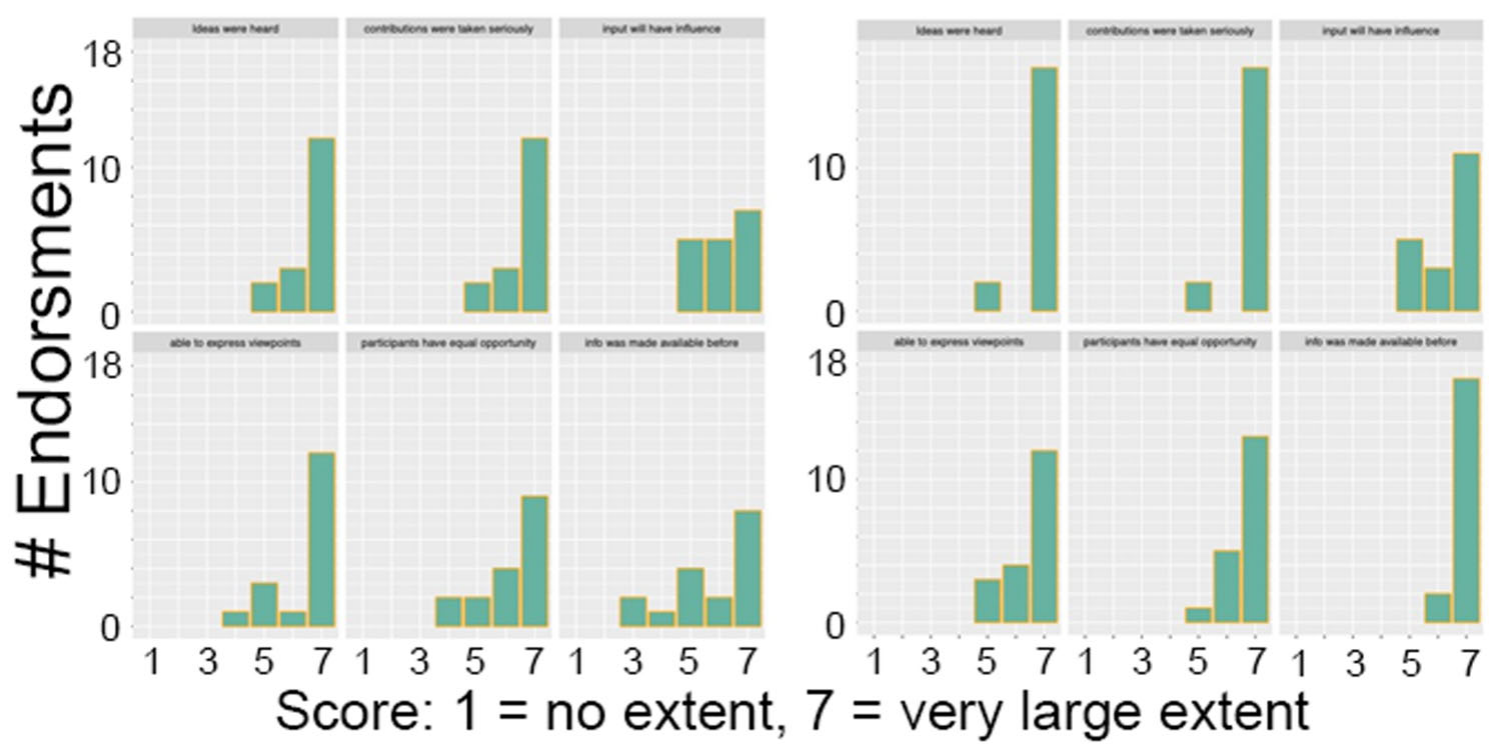
IMAPCT PEET results—November 2023 (left) and May 2025 (right). Participants completed the PEET after each project team and focus group meeting. Bars in the graph represent the number of participant endorsements for a given answer category, scored on a 7‐point Likert scale (1 = “to no extent,” 7 = “to a very large extent”). The left graph shows data from November 2023 whereas the right shows the results from March 2025. Improvements were observed across all domains, particularly in participant perceptions of influence, equal opportunity to contribute, and availability of information prior to activities. IMPACT, Identifying research targets by Merging Patient And Clinician Treatment information; PEET, patient engagement evaluation tool.

As supportive infrastructure to facilitate PFIC specific CER, we developed a centrally aggregated, readily accessible, and easily adaptable web‐based platform—the TEA (https://impacttea.pfic.org). TEA collates and displays relevant published research translated into lay language as well as information regarding patients’ experience with the most prevalent treatments and interventions. To inform TEA design and applicability, data from five focus groups that included 50 patients/parents and 10 clinicians (from seven unique academic centers) were included. Following iterative feedback from stakeholder focus groups, TEA design included three “treatment paths” (1) medications, (2) surgical interventions, and (3) vitamins and nutrition (Table 1). Patient‐level experience data are then collated and disseminated via the TEA in a “community experiences” section highlighting both the “benefits” and “burdens” of each therapy as experienced by the PN community as well as “impacted areas of life” (Figure 2). Ultimately, the TEA represents a dynamic platform that has been designed to iteratively engage with the community, collate experiences, and display information. To date, there have been 102 total entries across all treatment pathways.

**Table 1:** Treatment experience app treatment path information.

Medications 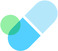	Surgical interventions 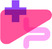	Vitamins and nutrition 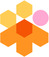
Antipruritics (bile acid modulators) o Cholestyramine o IBAT Inhibitors o Rifampicin o Ursodiol Antipruritics (non‐bile acid modulators) o Antihistamine o Carbamazepine o Naltrexone o Ondansetron o Sertraline Other o Blood Filtering systems (MARS, plasmapheresis) o Gabapentin o Loperamide o Sodium Phenylbutyrate	Cholecysto‐appendicostomy Ileal Exclusion Liver Transplant PEBD PIBD PTBD	Cyproheptadine MCT‐based Formulas/Oils NG Tube TPN Vitamin A Vitamin D Vitamin E Vitamin K

Abbreviations: IBAT, ileal bile acid transporter; MARS, molecular absorbent recirculating system; MCT, medium chain triglycerides; NG, nasogastric; PEBD, partial external biliary diversion; PIBD, partial internal biliary diversion; PTBD, percutaneous transhepatic biliary drainage; TPN, total parenteral nutrition.

### 3.3 IMPACT patient‐centered CER roadmap

Six education modules, each with a specific theme related to PFIC CER. (Supporting Information S1: Section S4) informed development of the IMPACT Roadmap (https://impactroadmap.pfic.org), a manual for developing and implementing patient‐centered CER projects in PN and more broadly within the PFIC community. The IMPACT Roadmap consists of three broad sections:

#### 3.3.1 IMPACT roadmap part 1—Engagement guidance

This section provides our guidance on (1) identifying barriers and solutions to patient‐researcher partnerships, (2) forming successful multi‐stakeholder PFIC research teams, and (3) developing communication and dissemination strategies. Within each category, stakeholder‐influenced recommendations can be found related to strategies to identify core leadership team stakeholders for each project, to support such teams, and to effectively motivate and sustain partnerships over time. Regarding dissemination, IMPACT found that participants preferred more traditional email, social media, and website‐based updates over more academically focused publications and print mail (Supporting Information S1: Section S5).

#### 3.3.2 IMPACT roadmap part 2—Future direction for PFIC CER

Recognizing future patient‐centered CER efforts would need to be grounded in values that reflected the collective parent and patient stakeholder voice, guiding principles were defined to act as mandates to the PN community in the planning and execution of research. We aimed to ensure that future efforts be outcome driven, patient‐centric, and transparent. The roadmap also outlines strategies for reducing the burden for study participation and considerations for the development of appropriate, patient‐centered CER questions in PFIC—specifically addressing variability in PFIC care and the implications of such variability on PFIC CER. Critically, using data collected from the TEA, focus groups, and surveys, IMPACT was able to define a list of community‐identified CER questions that require more research to improve care and decision‐making related to PFIC (Table 2) as well as outcomes, both patient‐reported and clinical, to prioritize in future efforts (Supporting Information S1: Section S5).

**Table 2:** PFIC patient‐centered CER priority questions.

Medications 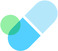	Surgical interventions 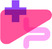	Vitamins and nutrition 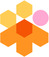
IBAT medications How do the different non‐IBAT medications compare in reducing itch? How does combining IBAT inhibitors with other medications compare to using IBAT inhibitors alone for reducing itch, gastrointestinal symptoms, and improving quality of life? What are the short and long‐term side effects of IBAT inhibitors versus external and/or internal biliary diversion surgery? Non‐IBAT medications How do alternative therapies like cold baths or massage impact itching and quality of life? What is the best medication to reduce PFIC‐related fatigue? What is the best way to manage the psychological effects of PFIC on patients and families during any stage of the disease? How does the mental health of PFIC patients receiving psychological intervention compare with those who receive none? Post liver transplant medications Which is more effective at reducing post‐transplant pruritus: IBAT inhibitors, non‐IBAT medications, or other treatments? What is the comparative effectiveness of antidiarrheals, IBAT inhibitors, external and internal biliary diversion surgery in reducing post‐transplant diarrhea? How do the short and long‐term side effects of post‐ liver transplant medications compare to pre‐ liver transplant medications?	Biliary diversion How does the likelihood of benefit from internal versus external biliary diversion vary among different PFIC types? What will work better to relieve itch—external or internal biliary diversion? What ostomy supplies have worked best with an external biliary diversion? Surgeries + IBAT Which delays the need for liver transplant longer: diversion surgery or IBAT inhibitors? How does the financial burden of surgical biliary diversion or liver transplant compare to the financial burden of being prescribed an IBAT inhibitor long‐term? How and when do you most effectively reverse an external or internal biliary diversion to solely using IBAT inhibitor? Liver transplant What are the best criteria to determine the necessity and urgency of liver transplant? When is the optimal time to pursue liver transplantation? How does a live donor liver transplant compare to a deceased donor liver transplant when it comes to care, longevity, and quality of life after transplant? How do growth and quality of life compare pre‐ and post‐ liver transplant?	Vitamins and supplements What types of OTC supplements best support growth and development? How do aggressive nutritional treatments (such as NG tubes or TPN) compare to more passive approaches (like oral formulas) in terms of tolerability, as well as their impact on weight, growth, and quality of life? How does regular versus infrequent monitoring of vitamin levels impact health and quality of life outcomes related to vitamin deficiencies? Diet Which is better: formula or blended food? How does an MCT‐enhanced diet impact growth versus a non‐MCT‐enhanced diet? How does a diet high in processed foods, sugars, fats, and starches compare with one containing fewer of these elements in terms of its impact on pruritus, gastrointestinal symptoms, quality of life, and liver health? Post transplant How do antidiarrheal medications (like loperamide) compare to external/internal biliary diversion surgery in reducing diarrhea after liver transplant?

Abbreviations: CER, comparative effectiveness research; IBAT, ileal bile acid transporter; MCT, medium chain triglycerides; NG, nasogastric; OTC, over the counter; PFIC, progressive familial intrahepatic cholestasis; TPN, total parenteral nutrition.

#### 3.3.3 IMPACT roadmap part 3 —Sustainability

Key to sustainability of this work is enabling broad use of the tools and resources that were built. Thus, public access to both the TEA and roadmap will enable lessons learned to be expanded by future efforts in PN and the broader rare disease community. Additionally, access to IMPACT educational modules via the PN website will enable continued CER‐specific learning for future participants, such as those newly diagnosed with a PFIC disorder.

### 3.4 Participant engagement

A comparison of PEET results from the start of the project in November 2023 to those collected at the end of May 2025 revealed a consistently strong and steadily improving level of engagement (Figure 2). Notable gains were observed in areas such as participants feeling their ideas were heard, having equal opportunity to contribute, and receiving relevant information before meetings. These improvements were driven by iterative adaptations made in response to the constructive feedback gathered through the PEET's open‐ended responses.

## 4 DISCUSSION

Patients and families affected by PFIC face a multitude of treatment decisions. These decisions often lack clear evidence on how choices will affect outcomes that are important to them—such as quality of life, daily functioning, or overall child health. PN families have previously shared experiences of navigating the health care realities of rare disease management, including the use of high‐risk procedures such as biliary diversion or liver transplant, without consistent guidance from their providers, expressing frustration at the absence of real‐world data to understand the benefits, burdens, and long‐term outcomes of various treatment options. This dialogue revealed a critical gap: the lack of patient‐centered engagement to identify and support informed decision‐making regarding treatment options and research prioritization for PFIC diseases. Patient‐centered CER offers a promising approach to address this challenge. It is vital, particularly in rare diseases such as PFIC, because it focuses on the experiences, needs, and priorities of patients and their families, rather than relying solely on the more traditional clinical and/or biological measures that, because of disease rarity and data scarcity, have failed to identify solid guidance or best practices. By engaging patients directly, patient‐centered CER ensures that studies address meaningful questions, improve care strategies, and guide decision‐making that aligns with what matters most to those living with the conditions. Project IMPACT was launched to provide the PFIC community with the necessary knowledge, and to create the necessary tools, to systemize treatment information and to establish a motivated base of patient families, clinicians, and researchers to enable future patient‐centered CER in PFIC.

Throughout the project, stakeholders participated in six virtual Learning/Feedback Modules where key patient‐centered CER concepts were introduced—such as the various roles of stakeholder partners, CER question development, and sustainability of multistakeholder research teams—and were reinforced through follow‐up surveys and focus group discussions. The modules established a shared foundation of knowledge across stakeholder groups while also generating valuable input that shaped both project deliverables and our ongoing engagement strategies.

To supplement the newly established CER knowledge and infrastructure within the PN community, the TEA was launched to support ongoing engagement, patient‐centered CER planning, and treatment education. The TEA was a novel engagement method designed to address a critical barrier that inspired project IMPACT: the lack of accessible, patient‐centered information about experiences with various PFIC treatment options. Without a shared understanding of treatments, many participants felt unprepared to engage their care teams about treatment priorities or contribute to discussions about CER opportunities. Developed with input from patients, parents, clinicians, and researchers, the TEA pairs clinician‐authored, plain‐language summaries with aggregated real‐world treatment experiences from patients and families. It has continued to enable community engagement via in‐app surveys, transforming it into a living platform for identifying real‐world management variability, relevant outcomes, and treatment comparators for future CER. Like the modules, it lowered barriers to participation and allowed meaningful contributions on participants’ own time. Data from the TEA have directly informed the final patient‐centered CER targets featured in the PFIC Roadmap and has created a lasting infrastructure for future CER planning. The TEA is actively maintained and updated by PFIC Network staff. New patient reports are routinely reviewed and incorporated into existing summaries of patient treatment experiences. In addition, periodic media campaigns are conducted to promote sustained engagement with the app.

A third critical output from IMPACT is the PFIC roadmap (https://impactroadmap.pfic.org/), designed to provide a structured, strategic framework that can be used by the PN community to guide research efforts towards meaningful, patient‐driven outcomes and help align stakeholders in their pursuit of shared goals and priorities. Specifically, the IMPACT roadmap offers guidance on forming multistakeholder research teams, choosing relevant and scientifically feasible CER questions, reducing study burden, and building long‐term engagement capacity in PFIC. It also includes practical tools ‐ such as detailed communication and dissemination strategies, and a 5‐year vision—and will serve as both a strategic planning tool and a living resource library to guide PN's ongoing efforts to build capacity for patient‐centered CER. The roadmap is also currently being used as a guiding framework for our follow‐up engagement project, which aims to establish a novel patient‐centered CER research consortium (Building Capacity to Engage in Patient‐Centered CER on Progressive Familial Intrahepatic Cholestasis [EACB‐42398]). As the project progresses, we anticipate updating the roadmap accordingly.

IMPACT and its key outputs have been shaped by input from the PFIC community—with improving PEET scores highlighting the positive engagement of our community and reflecting a strong indication of community readiness for CER. Importantly, many of IMPACT's core elements—such as engagement guidance, considerations for CER amid care variability common to rare diseases, and 5‐year plan to build and scale patent‐driven research infrastructure—are broadly applicable. Other rare disease patient advocacy groups could look to recapitulate the methods and success of IMPACT to create their own disease‐specific versions of key TEA and roadmap sections. We hope our efforts in IMPACT offer a practical, adaptable model for advancing patient‐centered CER across the rare disease landscape.

## 5 CONCLUSION

IMPACT successfully engaged key stakeholders from the PFIC community to build shared understanding of patient‐centered CER, define PFIC‐specific CER targets, and develop strategies for equitable and sustainable patient‐researcher partnerships. A crucial product of IMPACT is the TEA, an online platform to capture and share patients’ treatment experiences. Joint learning modules and focus groups resulted in an informed and enthusiastic group of community partners. These efforts culminated in a community‐designed roadmap outlining the infrastructure needed to design meaningful patient‐centered CER projects in PFIC as the next step.

## CONFLICT OF INTEREST STATEMENT

James E. Squires: Consulting for Ipsen and Mirum, participates as DSMB member for Sanofi. Emily Ventura: Consulting for Albireo, Mirum, and the Chan Zuckerberg Initiative in the past. Emily R. Perito: Consulting for Ipsen and Mirum, research grants to institution—Mirum, Gilead, Albireo. Laura Bull: Consulting for Ipsen. The remaining authors declare no conflicts of interest.

## Supporting information

Supporting File
