# Supplementary material for: Identifying patient‐centered outcomes in progressive familial intrahepatic cholestasis: Results from IMPACT
Source: J Pediatr Gastroenterol Nutr. 2026 May 21;83(2):215–22. doi: 10.1002/jpn3.70455 (PMC13446603; doi:10.1002/jpn3.70455)
Supplement: Supplementary file 1 [file JPN3-83-215-s001.docx]

**SUPPLEMENTAL MATERIAL**

**SECTION 1: PEET-6 Evaluation Tool and End-of-Year 1 / End-of Project Surveys**

**Patient Engagement Evaluation Tool (PEET-6)**

The Patient Engagement Evaluation Tool (PEET-6) developed by Moore et al. (J Clin Epidemiol. 2022;146:75–83) assesses six domains including trust, respect, fairness, competency, legitimacy, and accountability from a participant’s perspective. Each item is rated on a 7-point Likert scale ranging from 'No extent' (1) to 'Very large extent' (7). If a participant selected 'No extent,' an open-ended follow-up question invited qualitative feedback.

| **Item No.** | **Survey Question** |
| --- | --- |
| 1 | To what extent do you believe that your ideas were heard during the meeting? |
| 2 | To what extent do you believe organizers took your contributions during the meeting seriously? |
| 3 | To what extent do you believe that your input will influence final decisions that will be made in project IMPACT? |
| 4 | To what extent were you able to clearly express your viewpoints? |
| 5 | To what extent did all participants have equal opportunity to participate? |
| 6 | To what extent was information made available to you either prior or during the meeting so as to participate knowledgeably in the meeting? |

*Note: All six items were rated on a 7-point scale ranging from “no extent” to “very large extent; each item included an open-ended follow-up question to provide more specific feedback if one of the lower three response categories was selected.*

**IMPACT End of Year 1 Survey**

This survey was administered at the conclusion of Year 1 of project IMPACT to evaluate participant experiences with virtual learning modules, focus groups, and overall project outcomes.

| **Section** | **Survey Question** |
| --- | --- |
| Participant Info | Please enter today’s date. |
| Participant Info | Please indicate your role. |
| Learning Modules | Did you attend any of the virtual learning modules? |
|  | How would you rate the time commitment required for the IMPACT learning modules? |
|  | What did you think of the content of the IMPACT learning modules? |
|  | Do you think the compensation for the IMPACT learning modules was adequate? |
| Focus Groups | Did you attend any of the IMPACT focus group meetings in the past year? |
|  | How would you rate the time commitment required for the IMPACT focus groups? |
|  | What did you think of the content of the IMPACT focus groups? |
|  | Do you think the compensation for the IMPACT focus groups was adequate? |
| Year 1 Project Outcomes | The Treatment Experience App (TEA) is a great tool for PFIC families to learn and comment about PFIC treatments. |
|  | The learning modules helped me better understand patient-centered Comparative Effectiveness Research (CER). |
|  | The focus groups helped me better understand the challenges of comparing PFIC treatments. |
| Feedback | Which of the IMPACT project activities did you like most, and why? |
|  | Which of the IMPACT project activities should be improved, and how? |
|  | Do you have any additional comments or feedback? |

*Note:* *Survey questions included Likert-scale, categorical, and open-ended response formats; response scales contained between two and seven categories, with open-ended follow-ups inviting additional feedback if they selected one of the lower three response categories.*

**IMPACT End of Project Survey**

| **Section** | **Survey Question** |
| --- | --- |
| Collaboration (Patient Version) | When you think of Project IMPACT, what is the first activity, moment, or topic that comes to mind? Please write down whatever you think of first. |
|  | What is one new thing you did, learned, or felt more confident about because of IMPACT? |
|  | Do you feel more informed about PFIC treatment questions now compared to before participating in IMPACT? |
|  | Do you feel more comfortable discussing treatment options with your clinician or care team? |
|  | Do you think patients/families and clinicians/researchers have an equal voice in IMPACT? |
| Collaboration (Clinician/Researcher Version) | When you think of Project IMPACT, what is the first activity, moment, or topic that comes to mind? Please write down whatever you think of first. |
|  | What is one new thing you did, learned, or felt more confident about because of IMPACT? |
|  | Do you feel more informed about patients’ treatment questions now compared to before participating in IMPACT? |
|  | Do you feel more informed discussing treatment options with your patients? |
|  | Do you think patients/families and clinicians/researchers have an equal voice in IMPACT? |
| Communication and Materials | What percentage of project emails (e.g., meeting recaps and project updates) did you read since you started participating in project IMPACT? |
|  | What could we have done to make communications easier or more useful to read? |
|  | Were IMPACT meeting details (e.g., Zoom links, agendas) shared clearly and on time so that you could plan to attend? |
|  | Were project materials (e.g., recaps, summaries) easy to understand? |
| Future Involvement | How would you prefer to receive updates about future PFIC research activities and findings? |
|  | How likely are you to stay involved in future PFIC Network research initiatives (e.g., advisory roles, research teams)? |
|  | We recently applied for funding to continue the work we started in IMPACT. The goal is to build a research consortium  to help shape and support future PFIC patient-centered comparative effectiveness research. The planned project is  called PATH (PAtient-driven engagement for future comparative Treatment researcH). The work we proposed in PATH includes paid focus groups, committee roles, and other opportunities.  If PATH is funded, would you like to be contacted about opportunities to participate? |

*Note: Survey questions included Likert-scale, categorical, and open-ended response formats; response scales contained between two and five categories, with open-ended follow-ups inviting additional feedback if they selected one of the lower three response categories.*

**SUPPLEMENTAL MATERIAL SECTION 2: Treatment Experience App Data Collection Forms**

This survey was designed to collect patient- and caregiver-reported experiences with PFIC treatments, including medications, surgeries, and nutritional interventions.

| **Section** | **Survey Question** |
| --- | --- |
| Demographics/ Background | Where do you live? |
|  | What is your or your child's PFIC subtype? |
| Treatment Type & Timing | Would you like to share your experience with respect to a surgery, medication, or nutrition/vitamin? |
|  | Which surgery, medication, or nutrition/vitamin specifically? |
|  | Did you or your child receive this treatment? |
|  | When did you or your child receive this treatment? |
| Treatment Effects -Benefits & Burdens | How important was it for you to be able to receive this treatment? |
|  | Was this treatment chosen because of itch, malnutrition, or diarrhea? |
|  | Were there any side effects? |
| Treatment Effects -Sleep & Quality of Life Impacts | Did the treatment have an impact on the quality of sleep of the patient? |
|  | Did the treatment have an impact on the patient’s general quality of life? |
|  | Did the treatment have an impact on your family’s quality of life? |
| Delivery method, cost, & accessibility | What did you think of the delivery method of the treatment? By delivery method we mean route of administration (oral, injection, pill, liquid). (If you are filling out this survey about a surgery you can skip this question). |
|  | What did you think of the cost of this treatment in terms of financial burden? |
|  | Did you experience any problems getting access to this treatment? |
|  | Are there any other benefits or burdens that you would like to share? Please list them here, and also let us know how important these were or are for you. |
|  | Do you know of any treatments for PFIC and/or related diseases that you couldn’t find in the web app? If so, please list. |
|  | Did you or your child take or use any treatments that were not prescribed by a doctor and that you thought were helping? An example would be a specific nutrition item or over-the-counter treatment that you tried without a doctor  suggesting it. |

*Note: Survey questions included Likert-scale, categorical, and open-ended response formats; each treatment domain (e.g., itch, malnutrition, diarrhea) included parallel items assessing the extent of reduction (not at all, a little, a lot) and the perceived importance of that outcome (not important → somewhat important → extremely important).*

**SUPPLEMENTAL MATERIAL SECTION 3: IMPACT Participants**

**IMPACT Learning/Feedback Module Participation**

|  | **Number of participants** | |
| --- | --- | --- |
| **Module** | **Patient/parents** | **Clinician/researchers** |
| 1 | 29 | 8 |
| 2 | 19 | 6 |
| 3 | 17 | 5 |
| 4 | 20 | 3 |
| 5 | 31 | 5 |
| 6 | 16 | 5 |

**IMPACT Focus Group Participation**

|  | **Number of participants** | |
| --- | --- | --- |
| **Focus Group** | **Patient/parents** | **Clinician/researchers** |
| 1 | 7 | 4 |
| 2 | 12 | 5 |
| 3 | 8 | 5 |
| 4 | 13 | 5 |
| 5* | 35 | 35 |
| 6 | 10 | 3 |
| 7 | 11 | 6 |
| 8 | 12 | 3 |
| 9 | 10 | 2 |
| 10 | 13 | 4 |
| 11 | 10 | 4 |
| 12* | 16 | 11 |

**Focus group #5 was hosted as a hybrid session at the 2024 PFIC Family & Scientific Conference. Focus group #12 was hosted during an in-person breakout session at the 2025 Cholestatic Liver Disease Summit.*

**SUPPLEMENTAL MATERIAL SECTION 4: IMPACT Patient-Centered CER Module Themes/Topics and Questions**

- Module 1: What is Patient-Centered Comparative Clinical Effectiveness Research (CER)?
  - Introduced the core principles of patient-centered comparative clinical effectiveness research (previously referred to as “patient-centered outcomes research”, or “PCOR”).
  - (Includes both patient/family and clinician/researcher versions.)

| **Section** | **Survey Question** |
| --- | --- |
| Patient/Caregiver Version | Have you or your child ever been asked to participate in a research study about PFIC? |
|  | Have you or your child ever participated in a research study about PFIC? |
|  | When you enrolled your child or yourself in a research study (or if you were asked to do this), what factors were (or would be) important to your decision? |
|  | If you or your child did participate in a research study, did you ever find out what the results of that study were? |
|  | How did you learn about the results? |
|  | If you decided NOT to participate, how important were (or would be) the following barriers? |
|  | What are the most important questions that we need to  answer, or issues we need to address, to improve the  lives of PFIC patients? |
|  | To do research that is truly meaningful to PFIC  patients and their families, IMPACT must.... |
|  | To do research that is truly meaningful to PFIC  patients and their families, IMPACT should NOT... |
|  | What are your biggest worries about participating or  having your child participate in research? |
|  | In your opinion, what would research that is truly  "centered" on PFIC patients and their family be like? |
|  | What would make it easier or more appealing for you or  your child to participate in research projects? |
|  | Who are the key stakeholders that should be involved  in designing truly patient-centered research about  PFIC? |
|  | Demographics |
| Clinician/Researcher Version | Have you ever been involved in a clinical research study? |
|  | Have you ever been involved in a patient-centered outcomes research (PCOR) study? |
|  | What do you think are the main reasons that PFIC families agree to participate in research studies? |
|  | In your experience as a PFIC clinician/researcher, what do you think are the MAIN BARRIERS  for PFIC families to participate in research? |
|  | What are the most important questions that we need to  answer, or issues we need to address, to improve the lives of PFIC patients? |
|  | To do research that is truly meaningful to PFIC  patients and their families, IMPACT must.... |
|  | To do research that is truly meaningful to PFIC patients and their families, IMPACT should NOT... |
|  | Who are the key stakeholders-the voices we must  have-in truly patient-centered PFIC research ? Who  needs to be involved in designing these projects? |
|  | Demographics |

*Note: Survey questions included Likert-scale, categorical, and open-ended response formats for both patient/caregiver and clinician/researcher respondents.*

- Module 2: How Can I Contribute to Patient-Centered CER?
  - Explained the various roles patients, parents, and other stakeholders can play in shaping and conducting CER.
  - (Includes both patient/family and clinician/researcher versions.)

| **Section** | **Survey Question** |
| --- | --- |
| Patient/Caregiver Version | Tell us about a time when you and your current PFIC clinical care provider were truly working as partners. |
|  | Tell us about a time that you and your current PFIC clinical care provider were NOT working well as partners - what went wrong? |
|  | Imagine yourself (or other parents/caregivers, or patients) as a partner in a PFIC Network patient-centered outcomes research project - sharing your opinion in a meeting about research topics or the design of a new study. We don't want patient partner involvement to be just "checking a box" or a "rubber stamp." What could the research team do to help you feel like a true partner in these discussions? (Don't worry, you're not committing to anything right now - we are just collecting ideas about the best way to involve patients and families in this work). |
|  | The following list contains potentially important barriers that patients and their families may face to participate as a partner in designing and carrying out patient-centered research projects. Please indicate how important you think these potential barriers are for you. |
|  | What could we do to overcome those barriers - to shape meetings, teams, or projects to make sure that you and others are true partners in patient-centered outcomes research? |
|  | What do you think that families or patients could gain by participating in PFIC Network’s patient-centered outcomes research? |
|  | The videos you watched focus on adult patients. Our goal is for parents, caregivers, and pediatric patients to be partners in PFIC Network’s patient-centered outcomes research. What unique challenges will our child and family-focused projects face? |
|  | Based on this module, to do research that is truly meaningful to PFIC patients and their families, IMPACT should… |
|  | Based on this module, to do research that is truly meaningful to PFIC patients and their families, IMPACT should NOT… |
|  | Demographics |
| Clinician/Researcher Version | In your clinical work with PFIC patients, how do you form effective partnerships with patients and families? |
|  | What are the biggest barriers to forming partnerships with patients and families that you face during clinical care? |
|  | We don't want patient/parent involvement to be just "checking a box" or a "rubber stamp" in PFIC Network's future PCOR projects. How can we make sure that they are true partners in this work? |
|  | What do you think providers could gain by participating in PFIC Network's PCOR projects? |
|  | The following list contains potentially important barriers to participating in PCOR projects that providers and PFIC treatment centers may face. Please indicate how important you think these potential barriers are in getting PCOR projects underway in PFIC research. |
|  | What could we do to overcome these barriers? |
|  | The videos you watched focus on adult patients. Our goal is for parents, caregivers, and pediatric patients to be partners in PFIC Network’s patient-centered outcomes research. What unique challenges will our child and family-focused projects face? |
|  | Based on this module, to do research that is truly meaningful to PFIC patients and their families, IMPACT must… |
|  | Based on this module, to do research that is truly meaningful to PFIC patients and their families, IMPACT should NOT… |
|  | Demographics |

*Note: Survey questions included Likert-scale, categorical, and open-ended response formats for both patient/caregiver and clinician/researcher respondents.*

- Module 3: How Do We Develop Research Questions That Matter?
  - Provided guidance on framing research questions that reflect the priorities and of patients and families.
  - (Includes both patient/family and clinician/researcher versions.)

| **Section** | **Survey Question** |
| --- | --- |
| Patient/Caregiver Version | In your experience as a patient or parent/caretaker: when you and your provider are considering a PFIC treatment - a new medication for example, or a surgery such as liver transplant - what are the most important things for you to know about the treatment? |
|  | What are the most concerning risks/harms of the treatments that you or your child have received? |
|  | What are the most important outcomes of treatments that you or your child have received? |
|  | Based on our focus group discussions, the IMPACT team is currently exploring three priority areas for CER studies. Please choose two that you think are the most important to address. |
|  | Are there any areas that you think would be important to consider that we have not listed? |
|  | Which particular treatments do you think are feasible to compare? |
| Clinician/Researcher Version | In your experience as a clinician or researcher what are the most important outcomes that need to be achieved right after a PFIC diagnosis? Please specify by subtype/gene/protein if relevant. |
|  | What are the most concerning risks/harms of the treatments that you recommend or provide to PFIC patients directly after their diagnosis? Please specify by subtype/gene/protein if relevant. |
|  | What do you think are the main obstacles to reduce variability in care in PFIC? Please specify by subtype/gene/protein If relevant. |
|  | How do you think these obstacles can be overcome? |
|  | Based on our focus group discussions, the IMPACT team is currently exploring three priority areas for CER studies. Please choose two that you think are the most important to address. |
|  | Are there any areas that you think would be important to consider that were not listed? |
|  | Which particular treatments do you think are feasible to compare? |

*Note: Survey questions included open-ended and multiple-choice response formats for both patient/caregiver and clinician/researcher respondents.*

- Module 4: Virtual Tour of the Treatment Experience App (TEA)
  - Offered a walkthrough of the TEA platform and invited participants to give feedback on its design and usability.
  - (Includes both patient/family and clinician/researcher versions.)

| **Section** | **Survey Question** |
| --- | --- |
| Patient/Caregiver Version | Do you have any suggestions for how we can make the PFIC IMPACT Treatment Experience App (TEA) better? |
|  | Do you have any edits or additions to improve the lay summaries for existing treatments in the app? |
|  | Are there any PFIC treatments that you or your child have been prescribed that are missing from the TEA (e.g., medications, surgical interventions, or vitamins & nutrition)? |
|  | Do you have any questions or additional comments about the PFIC IMPACT Treatment Experience App? |
| Clinician/Researcher Version | Do you have any suggestions for how we can make the PFIC IMPACT Treatment Experience App (TEA) better? |
|  | Would you recommend any edits or additions to improve the clinical or lay summaries for existing treatments in the app? |
|  | Are there any PFIC treatments you have prescribed that are missing from the TEA? (i.e., medications, surgical interventions, or vitamins & nutrition) |
|  | Do you have any questions or additional comments about the PFIC IMPACT Treatment Experience App? |

*Note: All survey questions used open-ended response formats for both patient/caregiver and clinician/researcher respondents.*

- Module 5: Review of Draft PFIC Patient-Centered CER Targets
  - Collected input from participants on the draft list of PFIC patient-centered CER targets.
  - (Includes both patient/family and clinician/researcher versions.)

| **Section** | **Survey Question** |
| --- | --- |
| Patient/Caregiver Version - Background Information | What is the patient's age? |
|  | What is the patient’s gender? |
|  | What is the patient’s diagnosis? |
|  | Has the patient received a liver transplant? |
|  | Has the patient received an IBAT inhibitor (e.g., maralixibat or odevixibat)? |
| Patient/Caregiver Version - Treatments | Background: In previous IMPACT modules and focus groups, we've talked about the dilemmas and uncertainties patients are facing in their everyday clinical care. We began compiling a list of patient questions that could be transformed into comparative effectiveness research, including: - What is the comparative effectiveness of the different non-IBAT medications in reducing itch? - How does combining non-IBAT medications with IBAT inhibitors compare to using IBAT inhibitors alone for reducing itching, improving gastrointestinal symptoms, and quality of life? - How do different surgeries (external or internal biliary diversion, and liver transplant) compare in improving the quality of life for PFIC patients and their families? - How do aggressive nutritional treatments (like NG tubes or TPN) compare to more passive approaches (like oral formulas) in improving weight, growth, and quality of life in PFIC patients? - How do the different types of aggressive nutritional treatments compare in tolerability and in improving weight, growth, and quality of life? - How does care with a dietitian compare to care without one when it comes to better nutrition and overall well-being? - How does a high-fat diet compare to a low-fat diet in managing gut symptoms, itching, and quality of life for PFIC patients? - How do anti-diarrheal medications (like loperamide) compare to biliary diversion  surgery in reducing diarrhea after a liver transplant? We want to make this list as complete as possible and need your  help to figure out what's missing!  Question: What other questions have you had about managing PFIC that aren't on this list? Think back to all the moments when you needed answers-whether it was right after diagnosis, in the years since, or now. What were the things you asked your doctor about but didn't get the answers to that would have made your life less difficult or less stressful? Your questions can be about anything that impacts life with PFIC, big or small - whether it's about treatments, daily treatment-related challenges, or even something as specific as how certain foods might affect symptoms. Don't hold back - your ideas can help us uncover what's missing! |
| Patient/Caregiver Version - Outcomes | Background: In previous IMPACT modules and focus groups, we've talked about the outcomes that are most important to PFIC patients' and their families' everyday lives. Outcomes are the changes that occur because of a treatment or care approach, such as less itch or better sleep. Outcomes are also how we measure meaningful differences between treatments. For example, we can compare two treatments based on how effectively each one reduces itch. Most research studies measure multiple outcomes to learn as much as possible. So far, we know that the following outcomes matter to PFIC patients and families: - Itch relief - Improved quality of life - Improved sleep quality – Growth (height) - Weight gain - Reduction of diarrhea - Lowered financial burden of disease - Reduced abdominal pain and discomfort - Slowed/halted disease progression - Maintaining optimal vitamin levels - Reduced hearing loss - Improved palatability (e.g., treatment taste, ease of use)  Question: What other outcomes matter most to you and your family that we haven't listed yet? Please think about all the ways living with PFIC has affected you or your child's health, happiness, daily life, work, school, finances, and relationships. What ways has PFIC made life more difficult and/or stressful?  Were there any symptoms or related factors that, if addressed, would make you or your child's life better? Feel free to think outside the box-your unique experiences will help fill in what's missing! |
| Patient/Caregiver Version - Study Design | Background: Once we've gathered our lists of treatment questions and important outcomes, our next step will be to design a research study to try and find answers. In earlier IMPACT modules and focus groups, we talked about how the relevance of treatments and outcomes can vary depending on factors like PFIC subtype, transplant status, or other individual circumstances. We also explored study ideas that could apply to (almost) everyone, such as comparing itch treatments or nutrition interventions.  Question: Earlier in this survey, we asked for information about your subtype, transplant status, and other factors. We'd like to understand what type of research study feels most meaningful to you based on your journey with PFIC. For example, you could have a specific question related to being post-transplant that only applies to you and others in a similar situation. Would you prefer a first research study that addresses questions for the whole PFIC community, or one focused on a specific subtype, post-transplant care, or age group? Your input will help us better understand how to approach designing a study. |
| Patient/Caregiver Version - Participation | Background: Enrolling enough participants in a study is key to ensure the study results are convincing. In our most recent IMPACT focus group, we discussed ways to design a PC-CER study that would encourage enough patients and families to participate. Members shared ideas like: - Reducing the number of hospital visits required - Offering services at home or through local clinics - Minimizing medical trauma and requiring as little follow-up as possible - Using less invasive measures, like painless blood draw devices - Ensuring compassionate care from trial nurses - Providing compensation for participants, whether children or adults - Offering comforts, like toys for kids or therapy dogs  Question: What else would motivate you to participate in a research study comparing treatments for PFIC? Do you have any other ideas how to make participation in a study the least burdensome? |
| Clinician/Researcher Version - Background Information | What is your primary role? |
|  | Approximately how many PFIC patients have you seen in the past year? |
|  | Do you treat or care for PFIC patients directly? |
|  | Have you been involved in PFIC-related research as a PI or collaborator? |
|  | Which PFIC types do you typically work with (research or clinical setting)? |
|  | What age ranges of PFIC patients did you typically work with (research or clinical setting)? |
| Clinician/Researcher Version - Treatments | (Same background as patient/caregiver version - treatments)  Question: When thinking about treatment questions that can be addressed in a study, which ones do you think would be most relevant for PFIC patients and families?  If you are a clinician or nurse, think about the moments when your patients were seeking guidance-whether right after diagnosis, during follow-up visits, or in their current care. Which questions stood out as especially challenging or significant? These could range from treatment options to everyday quality of life concerns.  If you are a researcher, think about (side) questions that came up during your research. Your input can help us identify the gaps that matter most! |
| Clinician/Researcher Version - Outcomes | (Same background as patient/caregiver version - outcomes)  Question: What other outcomes do you think might be important to patients?  If you are a clinician or nurse, consider how your PFIC patient has shared the impact of the disease or treatments on their health, well-being, daily life, work, school, finances, or relationships. Which outcomes have been particularly significant or meaningful to your patients? Which outcomes have been most notable when assessing treatments or care strategies?  If you are a researcher, think about outcomes that you think should be included in a study. |
| Clinician/Researcher Version - Study Design | (Same background as patient/caregiver version – study design)  Question: We'd like to understand what type of research study feels most meaningful to you based on your own experience treating or researching PFIC. Would you prefer a study that addresses questions for the whole PFIC community, or one focused on a specific subtype, post-transplant care, or age group? Your input will help us better understand how to approach designing a study. |
| Clinician/Researcher Version - Participation | (Same background as patient/caregiver version – participation)  Question: Do you have any other ideas how to make participation in a study the least burdensome for patients? |

*Note: Survey questions included open-ended and multiple-choice response formats for both patient/caregiver and clinician/researcher respondents.*

- Module 6: Multistakeholder Research Teams
  - Examined how to build successful multistakeholder teams capable of planning and carrying out CER projects.
  - (Includes both patient/family and clinician/researcher versions.)

| Section | Survey Question |
| --- | --- |
| Patient/Caregiver Version - Readiness & Support Needs | How important would the following types of support or resources be in helping you feel comfortable contributing to a research team? |
|  | What would make participation in a multi-stakeholder research team attractive to you? |
|  | How confident do you feel about participating as a partner in a multi-stakeholder research team? |
| Patient/Caregiver Version - Motivations & Concerns | How motivating would the following factors be in your decision to participate in a research team? |
|  | What concerns do you have about working with researchers? |
|  | Which questions or concerns (if any) do you have about how PFIC patients and caregivers can contribute to research? |
| Patient/Caregiver Version - Qualities of a Successful Team | How important do you think the following qualities or practices are for a successful multi-stakeholder research team? |
| Clinician/Researcher Version - Readiness & Support Needs | How important would the following types of support or resources be in helping you feel comfortable working with patients and caregivers as research partners in a multistakeholder research team? |
|  | What would make participation in a multi-stakeholder research team attractive to you? |
|  | How confident do you feel about working with patients and caregivers as partners in a multi-stakeholder research team? |
| Clinician/Researcher Version - Motivations & Concerns | How motivating would the following factors be in your decision to participate in a multi-stakeholder research team? |
|  | What concerns do you have about working with patients and caregivers as research partners? |
|  | Which questions or concerns (if any) do you have about how PFIC patients and caregivers can contribute to research? |
| Clinician/Researcher Version - Qualities of a Successful Team | How important do you think the following qualities or practices are for a successful multi-stakeholder research team? |

*Note: Survey questions included Likert-scale and open-ended response formats for both patient/caregiver and clinician/researcher respondents.*

**SUPPLMENTAL MATERIAL SECTION 5: Dissemination Feedback**

When surveyed on preferences for receiving communications about research activities and findings, participants selected the responses listed in the table below.

**Supplemental Table 3. IMPACT Dissemination Preferences**

| **Response** | **Patient/parents** |
| --- | --- |
| Email | 16 |
| Website | 12 |
| Social media | 8 |
| Live Webinars | 6 |
| Print Mail | 5 |
| Academic Publications | 4 |
| Blog Posts | 2 |

Participant feedback indicated a strong preference for receiving information via email newsletters and websites.

**Community Identified Outcomes**

Priority Patient Reported Outcomes:

*Medication Specific*:

- Itch relief, skin health, sleep quality, reduction of pain, reduction of fatigue, reduction of diarrhea, reduced frequency and severity of episodes

*Surgery Specific*:

- Itch relief, reduced abdominal pain/discomfort, improved social acceptance (e.g., concerning ostomy bags, growth delays), reduction of diarrhea, improved toilet training

*Nutrition Specific*:

- Improved palatability (e.g. treatment taste, ease of consuming quantity)

*Cross Intervention*:

- Improved patient quality of life, improved quality of life for caregivers/family/siblings, improved mental health (reduced anxiety/depression/irritability), improved focus and school performance, improved ability to enjoy major life events, play and swim

*Care Delivery and Support Specific*:

- Reduced number of workdays missed for care, lowered financial burden of the disease and treatments, impact of delayed insurance approvals on quality of life, impact of switching insurance companies on treatment access, improved ease of acquiring compounded medications, trust in provider/healthcare system, improved testing for cancers

Priority Clinical Outcomes:

*Medication Specific*:

- Slowed/halted disease progression, native liver survival

*Surgery Specific*:

- Slowed/halted disease progression, native liver survival

*Nutrition Specific*:

- Improved vitamin levels, weight gain, improved growth (height), improved strength, reduction/prevention of hearing loss

**SUPPLEMENTAL** **FIGURE 1**
